# Multilevel analysis on prevalence and associated factors of modern contraceptive uptake in Somaliland: based on The Somaliland Health and Demographic Survey 2020

**DOI:** 10.1186/s12978-024-01786-1

**Published:** 2024-05-21

**Authors:** Teshome Gensa Geta, Saad Ahmed Abdiwali, Mustafa Mohamoud Farah, Dereje Zewdu Assefa, Temesgen Tantu Arusi

**Affiliations:** 1 Department of Public Health, College of Medicine and Health Science, Wolkite University, Wolkite, Ethiopia; 2 Department of Public Health, College of Health Science and Medicine, Gollis University, Hargeisa, Somaliland; 3 Department of Anaesthesia, College of Health Science and Medicine, Wolkite University, Wolkite, Ethiopia; 4 Department of Obstetrics and Gynaecology, College of Health Science and Medicine, Wolkite University, Wolkite, Ethiopia

**Keywords:** Modern contraceptive method, Associated factors, Prevalence, Reproductive age, Somaliland

## Abstract

**Introduction:**

Contraception is the deliberate prevention of unwanted pregnancy through various contraceptive methods. Its uptake is low in Sub-Saharan African countries, particularly in east Africa. This might be linked to the high prevalence of unwanted pregnancies and the high fertility rate in the area. Although studies reporting the prevalence and associated factors of modern contraceptive uptake are available in other African countries, no study has been conducted in Somaliland. Therefore, the current study aimed to assess its prevalence and associated factors in Somaliland using Somaliland Health and Demographic Survey (SLHDS) data.

**Methods and materials:**

The study used Somaliland Demographic Health Survey (SLDHS) 2020 data. The survey was a national-level survey using a cross-sectional study design. A total of 3656 reproductive-age women were included in the current study. To determine independent predictors of modern contraceptive uptake, a multi-level multivariable logistic regression analysis was done. Random effect analysis, standard error (SE) and intra-cluster correlation (ICC) were computed.

**Results:**

The proportion of modern contraceptive uptake among reproductive age groups in Somaliland is 1%. Modern contraceptive uptake is significantly associated with the residence, educational level and wealth index of participants. Women from nomadic communities had lower odds (AOR: 0.25; 95% CI: 0.10, 0.66) of modern contraceptive uptake compared to those from urban areas. Being in the highest wealth quintiles (AOR: 17.22; 95% CI: 1.99, 155.92) and having a tertiary educational level (AOR: 2.11; 95% CI: 1.29, 9.11) had higher odds of using the modern contractive method compared to those with the lowest wealth quintiles and non-formal education, respectively.

**Conclusion:**

The prevalence of modern contraceptive uptake in Somaliland was very low. It is associated with the level of education, wealth index and residence of the women.

## Introduction

Contraception is the deliberate prevention of unwanted pregnancy through various contraceptive methods. It can be a traditional and/or modern contraceptive method [1]. Traditional contraceptive methods include the Coitus Interrupts or withdrawal method, the lactational amenorrhea method and the rhythm method. The modern contraceptive methods include emergency contraceptive pills, oral ccontraceptive pills, injectables, intrauterine contraceptive devices (IUCD), condoms, diaphragm, spermicides and female sterilization (Tubal ligation) [2].

Globally, out of 1.9 billion women in the reproductive age group, around 874 million women use modern contraceptive methods. Women need to use a contraceptive method and get satisfied with modern contraception were 77% globally. Its prevalence was lowest in the sub-Saharan Africa region (56%) compared to other regions [3]. The East African countries were among the lowest rate of uptake recorded. The report from a multi-country analysis of DHS in East Africa showed a very low pooled prevalence (20.68%) [4].

According to Sustainable Development Goal 3 (SDG 3), it was planned to increase universal access to reproductive health services, including family planning [5] to ensure that every pregnancy is wanted and planned. Despite the plan, uptake of modern contraceptive methods is still low in sub-Saharan African countries and have a high fertility rate compared to other regions [6]. Somali in east Africa is the country with high fertility rate (6.9 children per women). The 2020 Somali Health and Demographic survey (SHDS) indicated that 7% of sexually active women using contraceptive method and among them only 1% were using modern contraceptives [7]. It is the lowest modern contraceptive uptake region report by world contraceptive use 2022 by the UN [8]. This low uptake of modern contraceptive methods contributed to the high prevalence of unwanted pregnancies and interns contributed to a high proportion of adverse pregnancy outcomes in African countries [9]. It has also negative impact on the socioeconomic well-being of the family, community and the nation at large [10].

There are many factors associated with not using the modern contraceptive method. It was indicated that community prohibition due to traditional and religious practice negatively affects the uptake of modern contraceptive methods [11, 12]. The study conducted in sub-Saharan countries showed socio-demographic factors including age, educational status, marital status, residence and wealth index were found to be associated with contraceptive uptake [13–15]. Evidence also shows that access to information and knowledge about modern contraceptive methods affects their uptake [16, 17].

Even though different studies conducted in Africa showed low uptake of modern contraceptive methods and their contributing factors, no study has been conducted on this subject in Somaliland. Hence, the current study aimed to assess the prevalence and determinant factors of modern contraceptive uptake using 2020 Somaliland Demographic and Health Survey data.

## Methods and materials

### Study area

The study was conducted in Somaliland, officially called the Republic of Somaliland. The country is an unrecognized de facto sovereign state in the horn of Africa. It has six geographical regions; Awdal, Marodijeh, Sahil, Togdheer, Sanaag and Sool. Its claimed territory has an area of 176,120 square kilometers with approximately 5.7 million residents as of 2021. Somaliland has several challenges regarding access to health care services, particularly in rural communities. However, the Essential Package of Health Services (EPHS), in line with the WHO building blocks of the health system, was developed by the Ministry of Health Development to improve the healthcare system at all levels. Health care services are delivered through five tiers: the community level, the primary health unit (PHU), the health center, the referral health center/district hospital, and regional hospitals [18].

### Study design and study period

A study used data from Somaliland Demographic and Health Survey (SLDHS) which is conducted by national-level survey in 2019 to assess factors associated with uptake of modern contraceptive method.

### Data

The Somaliland Demographic and Health Service (SLDHS) considered six geographic regions for strata and the residences (urban, rural and nomadic) during sampling. For urban and rural areas, Geographic Information System (GIS) software was used to select the enumeration area (EA). A total of 2,806 (1,869 in urban and 937 in rural) dwelling structures are formed for sampling frames. The selection of 35 Enumeration areas (EA) was done by probability proportional to the size of dwelling structures. Then, households were listed in 35 EAs and 10 primary sampling units (PSU) were selected from 35 EAs using a probability proportional sampling technique [19]. To construct a sampling frame for nomadic residents’ temporary nomadic settlements (TNS) were used. The list of TNS was considered as a sampling frame with an estimated number of households in each TNS being the measure of size. A total of 1,448 TNS dwelling structure was identified and the selection of EAs was done in the same way for urban and rural residents. The final sampling unit (households) was selected by systematic sampling techniques.

All ever married women aged 15–49 were eligible to be interviewed. Total of 6285 women were successfully interviewed. The current analysis concentrated on history of uptake of modern contraception and its associated factors. Therefore, including the subjects with outcome variable gives final sample of 3656 women. Data collection was conducted by trained interviewers using a structured interviewer-administered questionnaire via the CSPro Android platform [20]. Before survey data collection, training for supervisors and data collectors was given; and a pre-test was done. Data collection was closely supervised by supervisors and GPS tracking of field operations.

### Study variables

The dependent variable was the uptake of modern contraceptive methods which was dichotomized as those ever used and not used contraceptive methods. Independent variables were individual-level variables including; respondent’s age, educational level, wealth quantiles, marital status, listening to the radio, watching television, owning a mobile phone, ever used internet, intention for contraceptive use, heard about family planning on the radio, heard about the family on TV, heard about family planning by text message, get information from health professionals and also community level variables including residence and region were included in the current study.

### Data processing and analysis

The data was exported to STATA version 14.0 software for analysis. Descriptive statistics are computed by summary indices including mean, frequency and percentage. To assess the association of dependent variables with independent variables binary logistic regression analysis was done. Again, to determine independent predictors of modern contraceptive method multi-level multivariable logistic regression analysis was done to determine individual and community level factors in four models. Model I (null model) is without any explanatory variables, Model II contains individual-level factors, model III contains community-level variables and Model IV contains both individual-level and community-level factors. Random effect analysis, standard Error (SE) and intra-cluster correlation (ICC) were computed. The estimates were weighted to reflect the population. Finally, variables with adjusted odds ratios (AOR) of *p*-value less than 0.05 were declared as having significant associations.

## Results

### Socio-demographic background of the study participants

A total of 3656 women were included in the analysis of current study from SLDHS 2020 data. The age range of participant was 15 to 49 with mean (± SD), 31.19 (± 8.05). All the participants were Muslims and majority did not attend formal education, 3040 (83.2%) (Table 1).

**Table 1:** Socio-demographic characteristics of the study population, Somaliland, 2020

**Variables**	**Category**	**Frequency (n)**	**Percentage (%)**
Age group (years)	<= 20	256	7.0
	20–29	1316	36.0
	30–39	1343	36.7
	40–49	741	20.3
Residence	Nomadic	1301	35.6
	Rural	1180	32.1
	Urban	1175	32.2
Region	Sanaag	848	23.2
	Sool	738	20.2
	Togdheer	667	18.2
	Sahil	467	12.8
	Awdal	474	13.0
	Marodijeh	462	12.6
Educational attainment	No formal education	3040	83.2
	Primary education	451	12.3
	Secondary education	110	3.0
	Tertiary education	55	1.5
Marital status	Married and in union	3233	88.4
	Divorced	263	7.2
	Widowed	160	4.4
Wealth quintile	Lowest	1230	33.6
	Second	584	16.0
	Middle	431	11.8
	Fourth	606	16.6
	Highest	805	22.0
Listen to radio	At least once a week	146	4.0
	Less than once a week	45	1.2
	Not at all	3465	94.8
Watching Television	At least once a week	416	11.4
	Less than once a week	100	2.7
	Not at all	3140	85.9
Owns Mobile Telephone	Yes	2763	75.6
	No	893	24.4
Ever used internet	Yes	309	8.5
	No	3347	91.5

### Information on contraceptive method

Majority of participants, 2245(90.9%) had no intention to use contraceptive method. Regarding about information related to family planning, only 464 (12.8%) heard information form radio and 589 (16.3%) learned about family planning from health professionals (Table 2).

**Table 2:** Reproductive history of the study participants, Somaliland, 2020

**Variables**	**Category**	**Frequency (n)**	**Percentage (%)**
Intention for contraceptive use (*n* = 2471)	Intended	226	9.1
	Not intended	2245	90.9
Heard about FP on radio (*n* = 3622)	Yes	464	12.8
	No	3158	86.2
Heard about FP on TV (*n* = 3622)	Yes	500	13.7
	No	3122	85.4
Heard about FP by mobile phone text message (*n* = 3622)	Yes	272	7.5
	No	3350	92.5
Health professionals talked about FP (*n* = 3622)	Yes	589	16.3
	No	3033	83.7

### Modern contraceptive method uptake

**Fig. 1:**
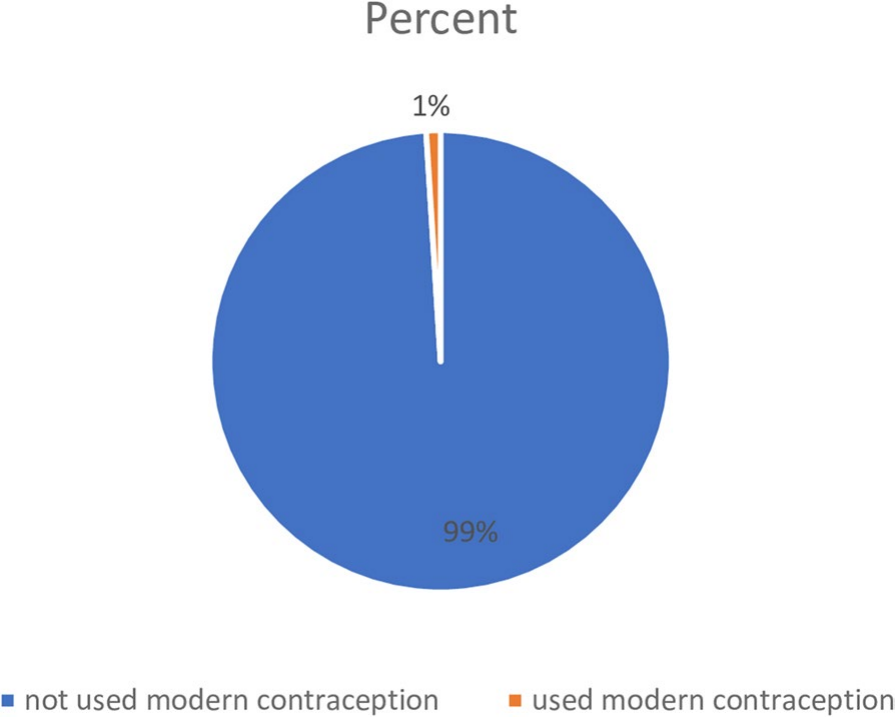
Proportion of modern contraceptive uptake in Somaliland, data from Somaliland Demographic and Health Survey (SLDHS), 2020

The proportion of modern contraceptive uptake among reproductive age group in Somaliland is 1%. The remaining, 3618 (99.0%) women had no history of taking modern contraceptive method (Fig. 1).

### Bivariate logistic regression analysis of factors associated with contraceptive uptake

Bivariate logistic regression showed women living in rural and nomadic residence had significant less odds of using modern contraceptive method compared to those living in urban community. Women from nomadic village had 76% less odds of using modern contraceptive method compared to urban women (COR = 0.23; 95% CI: 0.09, 0.57) (*p*-value = 0.002). Regarding women’s educational level, women with no formal education had 92% lesser odds of using modern contraceptive method compared to those attained tertiary level education (COR = 0.08, 95% CI: 0.03, 0.24) (*p*-value < 0.001) (Table 3).

**Table 3:** Bivariate logistic regression analysis of factors associated with contraceptive uptake in Somaliland, data from SLDHS 2020

**Variables**	**Category**	**Crude Odds Ratio (95% CI)**	***P*-value**
Age group	<= 20	-	-
	20–29	2.25 (0.75- 6.77)	0.147
	30–39	2.48 (0.84, 7.38)	0.100
	>= 40	1 (ref)	
Residence	Urban	1 (ref)	
	Rural	0.38 (0.17, 0.84)	0.016*
	Nomadic	0.23 (0.09, 0.57)	0.002*
Level of education	No formal education	0.08 (0.03, 0.24)	< 0.001*
	Primary education	0.20 (0.05, 0.71)	0.031*
	Secondary education	1 (0.28, 3.48)	1.000
	Tertiary education	1 (ref)	
Wealth Index	Lowest	1(ref)	
	Second	5.30 (1.02, 27.41)	0.047*
	Middle	5.75 (1.05, 31.51)	0.044*
	Fourth	5.12 (0.98, 26.41)	0.052
	Highest	17.25 (4.04, 73.57)	< 0.001*
Heard FP by radio	Yes	1 (ref)	
	No	0.41 (0.19, 0.84)	0.015*
Heard FP by TV	Yes	1 (ref)	
	No	0.17 (0.09, 0.33)	< 0.001*
Heard FP from HP	Yes	1 (ref)	
	No	0.29 (0.15, 0.56)	< 0.001*
Read about FP from magazine	Yes	1 (ref)	
	No	0.14 (0.06, 0.35)	< 0.001*
Ever use internet	Yes	1 (ref)	
	No	0.13 (0.07, 0.26)	< 0.001*

*HP* Health professional, *FP* Family planning, *CI* Confidence interval, *Ref* Reference group

^*^Significant *p*- value

### Factors associated with modern contraceptive method uptake

Multi-level multivariable logistic regression analysis was applied to determine individual and community-level determinants of modern contraceptive uptake. Four models were applied for analysis. According to random effect analysis, model I was a null model with no variable which only observed intercept. The ICC in this model was 12.8%, which indicates the presence of intra-cluster variability contributing to community-level variables. Hence, multi-level analysis was recommended. In multilevel analysis, educational attainment is significantly associated with the uptake of modern contraceptive methods. The odds of using modern contraceptive method among women who reached secondary school was 3.71 times higher compared to those with no formal education (AOR = 3.71; 95% CI 1.21 to 8.92). Women from nomadic residence had 75% lower odds of using modern contraceptive methods compared to women from urban residence (AOR = 0.25; 95% CI 0.10, 0.66) (Table 4).

**Table 4:** Multi-level multivariable logistic regression models on individual and community-level factors associated with uptake of modern contraceptive methods in Somaliland based on data from SLDHS 2020

Variables and Category	Model I	Model II	Model III	Model IV
	Empty model	Individual level variables	Community level variables	Both individual and community level variables
		AOR (95% CI)	AOR (95% CI)	AOR (95% CI)
**Education level**				
No formal education		Ref		Ref
Primary school		1.07 (0.41, 2.84)		1.18 (0.44, 3.20)
Secondary school		3.71(1.21,11.30)*		3.72 (1.18, 11.66)*
Tertiary school		2.11 (1.12, 8.92)*		2.11 (1.20, 9.11)*
**Wealth index**				
Lower		Ref		Ref
Second		4.81 (0.91,25.36)		8.02 (1.41, 45.74)*
Middle		4.33 (0.75,24.79)		14.82 (1.51, 146.09)*
Fourth		3.01 (0.54,16.75)		10.02 (1.04, 96.08)*
Highest		5.67(1.11,28.95)*		17.22 (1.90, 155.92)*
**Use internet**				
Yes		Ref		Ref
No		0.56 (0.21, 1.47)		0.57 (0.27, 1.55)
**FP HE by radio**				
Yes		Ref		Ref
No		1.04(0.43, 2.52)		1.01 (0.42, 2.43)
FP HE By TV				
Yes		Ref		Ref
No		0.59 (0.24, 1.43)		0.65 (0.26, 1.63)
**FP on Magazine**				
Yes		Ref		Ref
No		0.61 (0.18, 2.03)		0.58 (0.17, 1.99)
FP HE from HP				
Yes		Ref		Ref
No		0.55 (0.24, 1.43)		0.54 (0.26, 1.11)
**Residence**				
Urban			Ref	Ref
Rural			0.36(0.16, 0.84)*	0.76 (0.31, 1.94)
Nomadic			0.25(0.10, 0.66)*	3.63 (0.63, 20.71)
**Region**				
Awdal			Ref	Ref
Marodijeh			1.43 (0.32, 6.28)	1.16 (0.25, 5.21)
Sahil			0.63 (0.15, 2.65)	0.65 (0.15, 2.87)
Togdher			0.24 (0.04, 1.15)	0.30 (0.05, 1.61)
Sool			0.56 (0.14, 2.10)	0.54 (0.13,2.27)
Sanaag			0.56 (0.15, 2.09)	0.54 (0.19, 2.11)
**Random effect**				
Community level Variance (SE)	0.48 (0.34)	0.11 (0.19)	0.23 (0.29)	0.23 (0.29)
ICC (%)	12.8%	3.1%	6.7%	6.5%

*FP* Family Planning, *HE* Health Education, *ref* Reference group, *HP* Health Professional, intra-cluster correlation, *CI* Confidence interval, *AOR* Adjusted odds ratio

^*^Significant *p*-value

## Discussion

A current study revealed that uptake of modern contraceptive methods is only 1%. This report is lower than studies conducted in Burundi (23.8%) [21], South East Ethiopia (20.8%) [22], Amhara Region, Ethiopia (46.9%) [23], Ghana (18%, 36.8%) [20, 24]. The possible explanation for this could be due to cultural and traditional practices that discourage the uptake of modern contraceptive methods. The study area has a deep-rooted perception that having a larger number of children is as blessing from God and consider having a big family gives happy life [25]. Hence, most women do not want to use contraceptive method. The difference in report might also be explained by inadequate reproductive health services in the area compared to other studies [26] and differences in the socio-demographic characteristics of study participants.

The current study also examined individual and community-level factors associated with modern contraceptive uptake. It revealed that the educational level of women, wealth index and place of residence were found to be associated with contraceptive use. Women tertiary education had two times higher odds of using modern contraceptive methods compared to those with no formal education. This is in agreement with previous studies [27–32]. This might be due to the fact that educated women have more understanding of reproductive health and autonomous decision-making power in family planning compared to non-educated women. It can also be due to the fact that more educated women have a better understanding of reproductive health education and more access to reproductive health information than less educated women. Literacy also increases trust in scientific explanation of the use of contraceptive methods [29].

Women with the highest wealth index were 5.67 times more likely to utilize contraceptive methods compared to those with lowest wealth index. This report is in agreement with reports from other studies [13, 31, 33, 34]. It also in line with another study conducted in East Africa using multi-country demographic and healthy survey data [4]. This might be due to financial constraint that limits access to contraceptive methods. On the other hand, those women from the highest wealth quintiles might be more educated and have occupation that positively affects the uptake of contraception.

Regarding the residents of the participants, those women from nomadic and rural areas had lower odds of using modern contraceptive methods compared to those from urban areas. This report is supported by other studies conducted in Guinea [35], Southern Ethiopia [36], Northeast Ethiopia [37], Tanzania [38], Uganda [39] and other Sub-Saharan African countries [14]. This could be due to the fact that the quality and accessibility of reproductive health services are much lower in rural and nomadic areas compared to urban areas. In addition to this, cultural beliefs related with having large number of children and not using contraceptive methods are stricter in rural and nomadic communities. Poor access to reproductive health information in remote areas could have contributed to a lower uptake of contraceptive methods.

### Strength and limitations of the study

The data used in the current study is a national demographic and health survey that is representative and generalizable to the whole population. The analysis was done using multilevel analysis that determined both individual and community-level factors. It is the first study in Somaliland; hence, the result is robust and would provide important information for policymakers. However, the study had some limitations. The survey was cross-sectional that might have had recall bias. Some important variables were not included in the analysis due to their missing in the SLDHS data set.

## Conclusion

The prevalence of modern contraceptive uptake in Somaliland was very low (1%). The variables, including level of education, wealth index and residences, were found to be associated with it. Governmental and non-governmental organizations working on family planning should focus on promoting the education of women particularly among rural residents and economically weak members of the community.

## Data Availability

The datasets used and/or analysed during the current study are available from the corresponding author on reasonable request.
